# The prevalence of sleep deprivation and its impact among medical officers in a tertiary hospital, a cross-sectional study from Malaysia

**DOI:** 10.1371/journal.pone.0306574

**Published:** 2024-08-29

**Authors:** Aqil M. Daher, Ismail Burud, Mehrdad Subair, Lily Mushahar, Law Jia Xin

**Affiliations:** 1 Department of Public Health and Community Medicine, School of Medicine,IMU University, Kuala Lumpur, Malaysia; 2 Department of Surgery, School of Medicine,IMU University, Clinical Campus, Seremban, Malaysia; 3 School of Postgraduate Studies,IMU University, Kuala Lumpur, Malaysia; 4 Department of Nephrology, Hospital Tuanku Ja’afar, Ministry of Health, Seremban, Malaysia; 5 Department of Surgery, Hospital Tuanku Ja’afar, Ministry of Health, Seremban, Malaysia; Universiti Malaya Fakulti Perubatan: University of Malaya Faculty of Medicine, MALAYSIA

## Abstract

Sleep deprivation (SD), defined as an inability to get a minimum of 7 hours of regular sleep at night is a serious health problem that impacts the performance of medical professionals. This study aims to determine the impact of sleep deprivation on perceived performance among medical officers (MOs). A cross-sectional study design involved 231 MOs from six disciplines in Hospital Tuanku Ja’afar, a tertiary center in the south of Malaysia. A self-administered questionnaire was introduced in the English language. The questionnaire involved the sociodemographic characteristics; job-related factors, and the Sleep Deprivation Impact Scale (SDIS). The SDIS is a 12-question scale, rated on a 5-point Likert scale from strongly disagree to strongly agree. A higher SDIS score reflected a higher impact of sleep deprivation. A total of 206 MOs returned the completed questionnaire yielding a response rate of 89.17%. The mean age of respondents was 31.68 (±3.49) years. Most of the respondents were female, of Malay ethnicity, and married. More than three-quarters (78.64%) reported sleep deprivation. Being less effective in communication and formulating diagnosis (3 (1.01) vs 2.5 (1.15),p = 0.005); taking longer time to do things (3.44 (1.07) vs 2.8 (1.34),p = 0.001); and feeling unsafe while driving (3.56 (1.25) vs 2.93 (1.55),p = 0.006) manifested significantly higher mean among sleep-deprived respondents. In conclusion, sleep deprivation is a prevalent problem; that adversely affects crucial functioning domains that may endanger patients and healthcare providers alike. Radical countermeasures are required to ensure satisfactory sleep duration and address areas jeopardizing MO safety.

## Introduction

Sleep is a crucial biological process that is essential for maintaining physical, cognitive, and emotional health. However, in today’s fast-paced society, it is rare to achieve satisfactory sleep duration for individuals of all ages and professions. Sleep deprivation (SD) is defined as an inability to obtain a minimum of 7 hours of regular sleep at night [1], which is a serious health problem that takes a toll on large numbers of the world’s population. There are multiple contributing factors to SD. Among those, socio-demographic factors are age, gender, marital status, children, caffeine consumption, smoking status, and alcohol consumption [2]. For instance, female hormonal fluctuation is predisposed to insomnia and hence sleep deprivation. Older age is associated with changes in sleeping patterns like reduced deep sleep and frequent awakening. caffeine consumption, and smoking both can act as stimulants that delay the onset of sleep. The occupational factors include job designation, working hours per week, and work timing [3]. Occupational roles that involve shift work or high stress levels, particularly those requiring staying awake during traditional sleeping hours may interfere with natural circadian rhythms and contribute to irregular sleep patterns resulting in sleep deprivation [4].

Long working hours and short-term shifts are normal in the medical profession, particularly among medical officers and these factors might have desensitized them to the significance of sleep. Medical officers are known to have long working hours with the possibility of 24 hours on calls and variable shift timings which shows that they might be inclined to SD [5].

The high proportion of American physicians (43.1%) who proclaimed that sufficient sleep is difficult to accomplish highlights the demanding nature of the medical profession coupled with high-stress levels which jeopardize their plans for work performance [6]. Saudi medical residents report even higher levels of SD reaching 85.9% which may signify the influence of cultural, systemic, and occupational factors [7]. House officers from Singapore report sleeping less than 6 hours on non-call nights in general hospitals, highlighting a serious well-being issue that affects productivity. National strategies for addressing this issue are mandatory to promote the well-being of healthcare workers [8].

Studies have shown that there is an increased risk of hypertension, diabetes, obesity, cardiac problems, and behavioral changes associated with lack of sleep which might be attributed to the impairment in metabolic function, emotional and psychological stress caused by SD [9]. Sleep deprivation (SD) contributes to reduced attention span, responsiveness, slow reaction times, impaired memory, and decision-making [10]. Pathophysiological attribution implicates the sensitivity of the prefrontal cortex area to sleep deprivation which is the centre for decision-making and problem-solving capacities [11].

A range of negative outcomes including burnout, and clinically significant medical errors were associated with sleep impairment [12–15]. With lack of sleep, brain connectivity might suffer resulting in poor communication, impaired memory, and learning process. High cortisol level during stressful conditions affects the hippocampal memory processing centre. When all these conditions supervene, doctors’ ability to deliver safe services will be jeopardized [16].

On the same note, SD impacted the cognitive performance of the surgeons’ laparoscopic skills in a simulated environment [17]. Reportedly, the impaired attention, cognition, mood, and motor skills due to sleep deprivation threaten performance and professionalism as well as patients’ safety necessitating a coordinated approach to tackle this problem [18, 19].

The changes in disease patterns brought up by the second epidemiological transition have imposed greater pressure on an already challenged Malaysian healthcare system which became apparent during the COVID-19 pandemic. While NCDs continue to be the major burden of disease globally and in Malaysia specifically, lack of awareness among the general population and delay in diagnosis adds pressure to the healthcare system. Malaysian healthcare system faces many challenges; High patient volume and staff shortage are the major factors affecting the delivery of equitable healthcare services in Malaysia [20]. The high burnout rate (39.1%) reported among MO officers in Malaysia during the pandemic [21] reflected the need for proper planning and resource management. The 2019 Malaysia doctor-population ratio was (2:1000) compared to a target of 2.2:1000 [22] calls for innovative health reforms and radical solutions to address these gaps.

The highly demanding job of medical officers is seen as a source of stress that may affect sleep duration and quality. Moreover, the negative impact of sleep deprivation on healthcare providers is well documented in terms of its effect on cognitive performance, psychomotor skills, communication skills, decision-making, patient safety, and physical and mental health. Adding to that, the perceived complexity of cases managed at tertiary hospitals requires higher acumen and time. Serious ethical issues are likely to be observed when SD is not addressed appropriately. While a plethora of literature documented the negative impact of SD on the functioning of MO in different countries, little is known about its impact of self-perceived impact on MO in Malaysia. To the best of our knowledge, no previous Malaysian study has assessed the impact of SD. The objective of this study was to assess the prevalence of SD and its perceived impact on performance and its associated factors among a sample of medical officers in a tertiary hospital in Malaysia. The results of the study could provide insight to stakeholders and health professionals to formulate effective solutions to tackle this problem and ensure the safety of the patients and doctors.

## Materials and methods

### Population

This was a cross-sectional study design involving medical officers who are working at Tuanku Ja’afar Hospital, Seremban, Malaysia. Tuanka Jaafar is a Tertiary referral hospital where MOs are on call daily likewise the specific departments doing on-calls. The choice of this hospital for data collection was because the hospital has a capacity of 1143 beds. There are 23 clinical and nonclinical specialties. It is a referral center for all district hospitals, health centres, and all private hospitals and clinics in the state of Negeri Sembilan state.

Medical officers from multi-discipline: surgical, medical, anaesthesia, orthopaedics, accident and emergency, paediatric and Obstetrics &Gynaecology departments were invited to participate in this study.

All the mentioned departments are involved in on-call duties daily. Consent was taken from the respective departments for participation. O&G department declined to be part of the study due to work overload and a busy schedule. All MOs except those with comorbidities (that affect sleep) were sampled from May to July 2022. Such comorbidities may include COPD, GERD, DM, diagnosis of psychiatric disorder, and chronic pain due to musculoskeletal conditions.

Different hospital departments face unique challenges and patient volume. The workload associated with the complexity of cases in a specific department provides an optimal situation for examining sleep deprivation and elucidating its perceived negative impact. As such departments with a high workload that requires daily on-calls for MOs were selected. These departments play an important role in delivering specialized services to the largest number of patients. Moreover, the anticipated response rate also influenced the selection, a department in which seamless communication is already established would help achieve the target sample size.

### Sample size calculation

The initial sample size was determined to be 193 using the below-shown formula for linear regression analysis by Sokal R, Rohlf F [23] with the assumption of a 0.2 correlation between variables at a significance level of 0.05. Applying the variance inflation formula to accommodate multiple comparisons, the calculated sample size became 201. To accommodate for a 10% non-response rate, an additional 20 respondents were required, and this brought the final sample size to 231. The choice of sample size formula was justified due to the numerical nature of the dependent variable which is the SD impact score.


$$n_{1}=\frac{{\left(Z_{1-\propto /2}+Z_{1-\beta }\right)}^{2}}{{C(r)}^{2}+3}\;n_{p}=\frac{n_{1}}{\left(1-R^{2}\right)}$$

The minimum required proportion of the sample calculated for each department is shown in Table 1.

**Table 1:** Sample size calculation for sampling procedure.

Department size		Proportion	Required size from the department	obtained
Medical	102	0.30	70	69
Anaesthesia	53	0.16	36	34
Surgical	51	0.15	35	33
Orthopaedic	48	0.14	33	31
Paediatrics	44	0.13	30	28
Emergency	38	0.11	26	11
Total	336	1	231	206

### Sampling method and data collection

Respondents were selected using a simple random sampling method. A list of MOs from respective departments was obtained. The participants were selected on a probability proportional to size basis. Computer computer-generated program was used to select participants from the list of available MOs within each department. Selected participants were approached and invited to participate in the study. The researcher distributed the questionnaires to MO during break time. The researcher explained the objective of the study, risks, and benefits of participation. MOs’ confidentiality and voluntary participation were ensured. Informed consent was obtained before handing the questionnaire to the selected respondents. The questionnaire was then collected upon completion.

The study was conducted in accordance with the World Medical Association’s Helsinki Declaration for Human Studies. The study was approved by the International Medical University ethics committee with reference number 4.3/JCM-237/2022. Furthermore, this study obtained approval from the Medical Research and Ethics Committee (MREC), Ministry of Health Malaysia with reference number NMRR ID-22-00509-U8Q(IIR). Written informed consent was obtained from each respondent which was witnessed by an MO.

### Data collection tool

The questionnaire of this study is composed of four parts. The first three parts cover the independent variables, and the last part represents the dependent variable.: (1) Sociodemographic characteristics which are age, gender, marital status, long-term medication use, exercise, caffeine consumption, smoking status, and alcohol consumption. For this study, caffeine consumption was defined as the consumption of at least one cup of a caffeine-containing product daily [24]. Alcohol consumption was defined as the regular consumption of an alcohol-containing beverage over the past 12 months [25]. Due to the time limitation for respondents to fill in the questionnaire amid busy daily schedules, data on exercise was collected based on the WHO cut-off value of 3 or more days a week without probing the participants on the type of physical activity [26]. (2) Job-related factors included the number of working hours, work timings (whether morning or night), the number of night shifts per week, the department in which participants are employed, and the number of sleeping hours per 24-hour period. (3) The Sleep Deprivation Impact Scale measures the self-perceived impact of sleep deprivation. It consists of 12 Likert scale questions rated from strongly agree (5) to strongly disagree (1) with a higher score reflecting a higher impact of SD on performance. The scale generally assesses mental status, communication, and the ability to perform tasks. No distinct subscales are prescribed. The total SD impact score is obtained by summing answers of all items [27]. The internal consistency of the scale in this study was 0.943. Unreported exploratory factor analysis showed that each questionnaire item is a distinct factor corresponding to the original factor structure. The questionnaire was introduced in English to a population who is proficient in English.

### Statistical analysis

All data were entered and analysed using the IBM SPSS v. 26 Software. Descriptive statistics were calculated for all variables. Categorical variables were presented with frequency and percentage, and numerical variables were described with mean and standard deviation. Independent samples t-test was to compare the mean sleep deprivation impact between two groups and One-way ANOVA was used for more than two groups comparison. Simple linear regression was used to identify potential variables to be included in the multiple linear regression model. Any variable with a p-value equal to or less than 0.2 was considered for the next analysis step. The enter multiple linear regression method was used to identify factors associated with the perceived impact of sleep deprivation. The enter MLR method was chosen based on the best-fitting model that explains the association. Six assumptions were tested to illustrate the suitability of multiple linear regression analysis which are (1) Normality of dependent variable, (2) absence of multicollinearity (3) absence of autocorrelation (4) absence of outliers (5) Normal distribution of residuals and (6) homoscedasticity (S1 File). The significance level was set at 0.05.

## Results

Table 2 depicts the distribution of sample characteristics. Out of the 231 distributed questionnaires, 206 were complete and returned yielding a response rate of 89.17%. Among the 206 participants’ data available for analysis, the mean age was 31.68(±3.49) years. Most of the respondents were female, of Malay ethnicity, and married. Income-wise, less than half earned 4850–7099 Malaysian Ringgit (MYR) monthly, and less than a third earned more than 7000 MYR. The vast majority were not taking any medication, and around 70% were regular caffeine consumers. A small proportion were alcohol consumers (13.59%), and a very small proportion were smokers (3.40%). Working hours categories were evenly distributed. The vast majority (82.52%) worked the morning shift. More than three-quarters (78.64%) reported sleep deprivation.

**Table 2:** Distribution of sociodemographic characteristics and job-related factors.

		n	%
Gender	Male	81	39.32
	Female	125	60.68
Race	Malay	123	59.71
	Chinese	23	11.17
	Indian	60	29.13
Marital Status	Single widowed	55	26.70
	Married	151	73.30
Income RM	1000–4849	40	19.51
	4850–7099	97	47.32
	7100–10959	56	27.32
	10960 or more	12	5.85
Long-term medication use	No	193	93.69
	Yes	13	6.31
Caffeine Consumption	No	68	33.01
	Yes	138	66.99
Alcohol consumption	No	178	86.41
	Yes	28	13.59
Exercise	Never	62	30.10
	<3 times a week	124	60.19
	3 and above a week	20	9.71
Department	Medical	69	33.50
	Anaesthesia	34	16.50
	Surgical	33	16.02
	Orthopaedic	31	15.05
	Paediatrics	28	13.59
	Emergency	11	5.34
Working hours/week	40–49	51	24.76
	50–59	54	26.21
	60–70	56	27.18
	> 70	45	21.84
Work timings	Mainly morning	170	82.52
	Equal morning & night	36	17.48
Sleep Deprivation	No	44	21.36
	Yes	162	78.64
Age Mean (SD)	31.68 (3.49)		

Table 3 illustrates the mean distribution for each question and the total SD impact score. It was observed that the mean for each SDIS question was higher for those who reported SD. Specifically, being less effective in communication and formulating diagnoses; ability to report discharged patients; and feeling unsafe while driving manifested significantly higher mean among sleep-deprived respondents.

**Table 3:** Mean Sleep deprivation impact by sleep duration.

Sleep deprivation Impact Scale	Sleep Deprivation		p
	No	Yes	
	Mean (SD)	Mean (SD)	
I am more irritable	3.27 (1.09)	3.44 (1.00)	0.321
I am less empathetic towards my patients	2.50 (1.15)	2.82 (1.06)	0.082
My ability to interact with colleagues is diminished	2.64 (1.1)	2.93 (1.02)	0.102
I am less effective at communicating with patients and families	2.48 (1.17)	2.95 (1.09)	0.012*
My ability to concentrate during morning patient rounds is diminished	2.84 (1.27)	3.19 (1.1)	0.077
My ability to retain information on a new patient is diminished	2.91 (1.25)	3.16 (1.06)	0.181
My motivation to learn is diminished	3.05 (1.31)	3.41 (1.18)	0.075
My desire to teach medical students is diminished	3.05 (1.31)	3.33 (1.12)	0.157
I am less effective at formulating diagnosis or making clinical judgments	2.5 (1.15)	3 (1.01)	0.005*
My ability to write appropriate discharge orders and prescriptions is diminished	2.27 (1.13)	2.69 (1.04)	0.023*
It takes me longer to do things	2.8 (1.34)	3.44 (1.07)	0.001*
I feel unsafe driving home	2.93 (1.55)	3.56 (1.25)	0.006*
Total Sleep Deprivation Impact score	33.22(1.87)	37.91(0.77)	0.009*

* p value is significant at 0.05

Table 4 shows factors that were significantly associated with SD impact. MOs from anaesthesia and paediatric were found to have significantly higher mean SD impact. The post-Hoc test showed that MOs from orthopaedic department are significantly different from anaesthesia MOs and paediatrics MOs (p = 0.006). The mean SD impact score has parallelly increased with increasing working hours per week. Those performing morning and night shifts had a higher mean SD impact score than those performing morning shifts only.

**Table 4:** Distribution of mean SD impact score by explanatory variables.

Factor	Mean (±SD)	P value	Post-hoc test
**Department**			
Paediatrics	41.43 (7.99)		
Anaesthesia	40.79 (8.37)		
Surgical	38.64 (7.92)		
Emergency	35.09 (13.72)	0.001 *	Anaesthesia vs, Orthopaedics p = 0.007
			Paediatrics vs Orthopedics, p = 0.006
Medical	34.93 (10.62)		
Orthopaedics	31.81 (13.01)		
**Working hours/week**			
40–49	29.63(11.11)	< 0.001 *	40–49 vs all others, p≤ 0.001
50–59	36.89(10.03)		50–59 vs 40–49, p = 0.001
60–70	40.41(9.69)		60–70 vs 40–49, p<0.001
> 70	40.84 (7.36)		> 70 vs 40–49, p<0.001
**Work timings**			
Mainly morning	36.18(10.61)	0.032 *	
Equal morning & night	40.36(10.15)		

* p-value is significant at 0.05

Table 5 shows factors significantly associated with SDI using the enter method multiple linear regression analysis. Variables entered initially into the model are Age, number of working hours, timing of work, SD, and departments (anesthesia, orthopedics, pediatrics, and internal medicine). Independent variables entered into the model are Age, gender, race, marital status, income, long-term medications, caffeine consumption, alcohol consumption, exercise, department, working hours per week, work timing, no. of calls per week, shift work, and sleep deprivation. Two factors were associated with perceived self-impact of sleep deprivation, these are sleep deprivation and number of working hours. The model showed adequate fitness (S1 File)

**Table 5:** Results of multiple linear regression.

	Coefficients		p	95.0% Confidence Interval for B		Collinearity Statistics
	B	Beta		Lower Bound	Upper Bound	VIF
Age	.022	.007	.914	-.379	.423	1.116
working hours per week	3.132	.321	.000*	1.789	4.475	1.219
work timings	1.766	.063	.373	-2.138	5.671	1.258
SD	3.529	.136	.038*	.203	6.854	1.063
Anesthesia	1.365	.048	.552	-3.148	5.878	1.607
Internal medicine	-.533	-.024	.782	-4.323	3.257	1.832
orthopedics	-3.227	-.109	.169	-7.837	1.384	1.556
pediatrics	3.756	.121	.112	-.882	8.394	1.446

* p value is significant at 0.05

R^2^ = 0.212

Dependent variable is Sleep Deprivation Impact score

## Discussion

The impact of sleep deprivation is well recognized among health professionals where it may threaten patient and doctor safety alike. Studies that reported the effect of sleep deprivation on perceived self-performance among Malaysian physicians are scarce. We are reporting data from a tertiary hospital in Malaysia that is aimed to shed light on this prevalent problem. Our findings showed that sleep deprivation affects domains of functioning namely taking longer time to do things, communicating with patients and families, driving safely, and making clinical judgment among others. The main factors associated with the higher perceived impact included long working hours and sleep deprivation.

The findings that more than 3 quarter of the respondents suffered SD is alarming and necessitates immediate action. It highlights the severity and significance of this problem in Malaysia and similar settings. Stakeholders may benefit from such evidence to address the problem in wider-scale studies that may incorporate qualitative data to understand MO perspectives on possible remedy.

This prevalence may reflect the workload incurred by MOs and the possibility of staff shortage due to public-private doctor migration. Moreover, the large number and complexity of cases managed at a public tertiary center might be another explanation. Other studies from Malaysia documented the impact of high workload among doctors in terms of high turnover [28], job satisfaction [29], and burnout [30]. Our results are in tandem with reports from the Middle East where acute SD reached 85.9% [7] and around 66% of residents who were selected from the American Medical Association database reported sleeping 6 hours on average or less [31]. The accordance of studies from diverse regions of the world like the USA, and the Middle East affirms the global nature of SD and the need for a holistic approach to curb it. It also signifies the role of occupational burden in the causation of SD. The mean score of SD impact is higher than the median of total absolute score indicating that the sample perceives an appreciable impact of sleep deprivation. MOs from anaesthesia department and paediatrics reported the highest impact of SD. It is well-acknowledged that anaesthesia is among the highly engaging speciality in which MOs may perform alternate day calls and sleep shorter hours [32]. The nature of work in anaesthesia involves taking care of critically ill patients in the ICU, and attending to major trauma, cardiac arrests, and surgical emergencies. Paediatricians similarly attended to neonatal emergencies and attended emergencies related to obstetrics and paediatrics [33]. In addition to duration, sleep quality is another factor that may contribute to poor functioning among MO [34]. Studies showed that sleep deprivation-related fatigue is a causative factor of errors among anaesthetists [35, 36]. Quantifying the impact of a problem is crucial for recommending appropriate remedial action. This study quantified the impact of SD and identified the department most affected. It points out that SD is prevalent among MOs from departments dealing with acute medical and surgical emergencies alongside the stress factors that arises from these environments. The findings that MOs who perform mixed morning and night shifts had a higher perceived impact of sleep deprivation came according to our expectations. The sleep cycle circadian system, centered in the suprachiasmatic nuclei of the anterior hypothalamus oscillates over a period of approximately 24 h and determines the rhythmicity of sleep/wake states with respect to its main synchronizer, the environmental light-dark cycle. Night shifts force physicians to work at the circadian peak of sleep propensity, which may jeopardize waking function, and rotating work shifts disrupt circadian alignment and sleep consolidation.

Sleep-deprived medical doctors suffered a reduction in attention and concentration and delayed response to stimuli due to actuate sleep deprivation [10]. Social life and family life were significantly affected besides the quality of sleep among Saudi healthcare professionals who perform night duties [37]. Naps may be an effective countermeasure, but when in the setting of sleep deprivation and/or increased circadian sleep propensity, sleep inertia may impair performance on awakening [19]. A study showed that the adverse mental health effects of sleep deprivation and associated stress among paramedics have improved following a switch from a 24-hour shift pattern to an eight-hour shift [38].

Feeling unsafe to drive home was found to register the highest impact among MO who suffered sleep deprivation in this study. Research indicates that approximately 17% of residents in emergency medicine and anesthesia reported at least one sleep-related crash during their training [39].

Other reports showed that falling asleep and accident rates was higher in those who worked five or more extended days making them prone to fall asleep while driving or stopped in traffic as reported in a study by Laura et al [40]. Drivers involved in sleep-related accidents often do not remember falling asleep but recall being sleepy before the accident and that their driving was potentially dangerous. Once the driver reaches the stage of fighting to stay awake, the only safe countermeasure is to stop driving and take a short nap [41]. The risk of motor vehicle accidents is hazardous not only to night shift workers but also to pedestrians and drivers.

The findings that some of the functioning domain is mainly affected by sleep deprivation are consistent with other studies. Studies showed that duration and quality of sleep are significantly correlated with poor cognitive function [42, 43]. Mood and cognition are mainly affected after night shift [44, 45].

The next affected domain of performance is the effective formulation of diagnosis and clinical judgment. This consequence is closely related to the impaired cognitive function experienced by MO with SD. As mentioned earlier, SD contributes to reduced attention span, responsiveness, slow reaction times, impaired memory, and decision-making (1). Committing medical errors was reported as a significant outcome of lack of sleep among resident medical doctors [13] and intensive care unit staff [14, 15]. SD impacted the cognitive performance of the surgeons’ laparoscopic skills in a simulated environment [17]. The impaired attention, cognition, mood, and motor skills due to sleep deprivation threaten performance and professionalism as well as patients’ safety [18, 19]. Reported death due to SD as a result of medical errors was seen in two studies that documented doubling errors by surgical residents in simulated laparoscopic surgery [46, 47].

Sleep-deprived medical officers in this study reported a higher negative impact on communication. Communication encompasses cognitive attributes mainly attention and language. It demonstrated how SD affects cognitive function. Individuals who suffer from SD are prone to irritability, agitation, and mood disturbances. These factors were found to impair the psychomotor function, hence communication [48]. The verbal communication, speech duration, and volume might not be affected compared to the cognitive component of formulating ideas and communicating them. MOs may resort to a selective communication process to avoid difficult patients.

MO are important members of the medical workforce in ensuring optimal health care delivery to the patient. Providing them with better working arrangements will contribute to the betterment of the health delivery system.

Some limitations are recognizable in this study. Firstly, the data comes from a single center, and does not include all disciplines e.g., Obstetrics and gynecology may limit the generality of the study. Nonetheless, the study could serve as a spotlight to address this problem. Secondly, the workload and complexity of cases managed at the tertiary level differ from those managed at the secondary center; this might overestimate the problem’s magnitude. Thirdly, other factors that may cause sleep deprivation like psychological and family problems, among others, were not captured in this study. Finally, self-reported measures don’t necessarily reflect the actual performance. Exploring medical errors and accidents would be helpful to provide direction to stakeholders.

The study, despite its limitations, adds valuable information to existing literature. It shows the high prevalence of SD among medical officers in a tertiary hospital and the domains affected by SD. The study findings draw some public health implications that stakeholders could utilize. It highlights the importance of ensuring the healthcare provider’s safety. Measures include adequate staffing, removing inequalities in human resources management, and the possible introduction of rest time during the working shift. Revising current policies and encouraging research to identify factors associated with poor function like psychological and family issues.

## Conclusions

SD is a prevalent problem amongst medical officers; it adversely affects crucial functioning domains that may endanger patients and healthcare providers alike. Feeling unsafe driving home, taking longer time to do things; clinical judgment, and communicating with patients are the most affected domains by SD. Follow-up studies on the impact of SD on medical errors and patient safety are required to inform policies to remedy this serious concern.

## Supporting information

S1 File Study datset. (XLSX)
