# Correction: The prevalence of sleep deprivation and its impact among medical officers in a tertiary hospital, a cross-sectional study from Malaysia

**DOI:** 10.1371/journal.pone.0310783

**Published:** 2024-09-16

**Authors:** Aqil M. Daher, Ismail Burud, Mehrdad Subair, Lily Mushahar, Law Jia Xian

The fifth author’s name is spelled incorrectly. The correct name is: Law Jia Xian. The correct citation is: Daher AM, Burud I, Subair M, Mushahar L, Xian LJ (2024) The prevalence of sleep deprivation and its impact among medical officers in a tertiary hospital, a cross-sectional study from Malaysia. PLOS ONE 19(8): e0306574. https://doi.org/10.1371/journal.pone.0306574.
